# Determination of Imprint Effects in Ferroelectrics from the Quantified Phase and Amplitude Response

**DOI:** 10.1021/acsaelm.4c00875

**Published:** 2024-09-16

**Authors:** Subhajit Pal, Emanuele Palladino, Haozhen Yuan, Muireann Anna de h-Óra, Judith L. MacManus-Driscoll, Jorge Ontaneda, Vivek Dwij, Vasant G. Sathe, Joe Briscoe

**Affiliations:** † School of Engineering & Materials Science, Queen Mary University of London, London E1 4NS, United Kingdom; ‡ Department of Materials Science & Metallurgy, University of Cambridge, 27 Charles Babbage Road, Cambridge CB3 0FS, United Kingdom; § UGC-DAE Consortium for Scientific Research, University Campus, Khandwa Road, Indore 452017, India

**Keywords:** piezoelectric, ferroelectric, piezoresponse force microscopy, Kelvin probe force microscopy, imprint effect

## Abstract

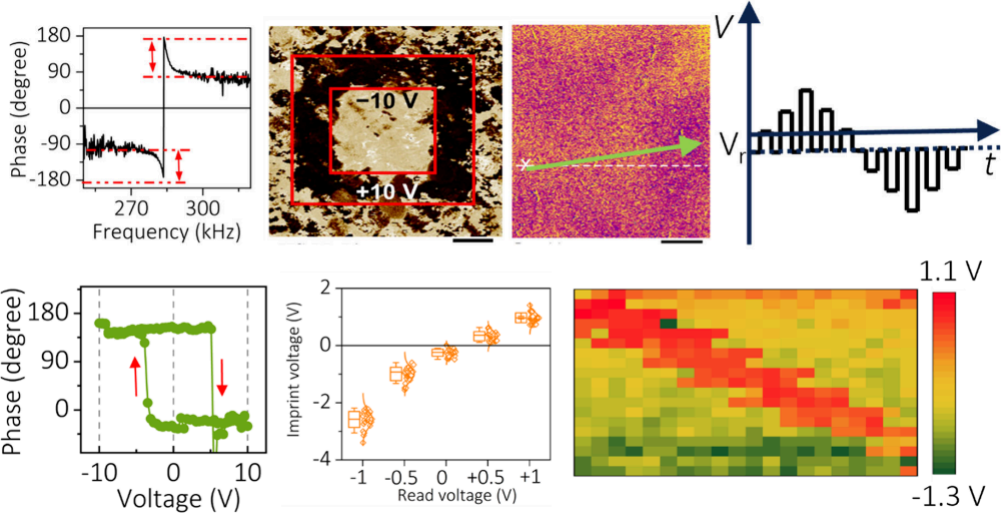

Piezoresponse force microscopy (PFM) is a robust characterization technique to explore ferroelectric properties at the nanoscale. However, the PFM signal can lead to misinterpretation of results due to the dominant electrostatic interaction between the tip and the sample. In this work, a detailed calibration process is presented and a procedure to identify the parasitic phase offset is demonstrated. To obtain artifact-free phase–amplitude loops, a methodology is developed by combining the outcomes from switching spectroscopy-PFM (SS-PFM) and Kelvin probe force microscopy (KPFM). It is demonstrated that the phase and amplitude loops obtained from SS-PFM at a specific read voltage, ascertained from the surface potential by KPFM, can convey accurate electromechanical information. These methodologies are applied to quantify the imprint voltage in BaTiO_3_ and BiFeO_3_, along with vertically aligned BaTiO_3_:Sm_2_O_3_ and BaTiO_3_:MgO nanocomposites. The variation of the imprint voltage measured under different tip voltages demonstrates the importance of selecting the correct read voltage in determining the local imprint voltage. Additionally, 2D imprint voltage maps in each domain of a BaTiO_3_ single crystal are obtained using the datacube-PFM technique, which allows pixel-by-pixel determination of artifact-free spatial variation of PFM phase–amplitude response.

## 1 Introduction

The quest for miniaturization of devices gives rise to the development of advanced characterization techniques. To this end, an electrical measuring unit combined with atomic force microscopy (AFM) results in advanced nanoscopic characterization techniques such as piezoresponse force microscopy (PFM),^1,2^ conducting probe-AFM (c-AFM),^3^ Kelvin probe force microscopy (KPFM),^4^ scanning electrochemical microscopy (SECM),^5^ and scanning thermal microscopy (SThM).^6,7^ Among these, PFM is considered to be a robust technique to investigate and manipulate ferroelectric domains and domain walls at the nanoscale.^1^ Combining PFM with other scanning probe techniques has resulted in the exploration of various functional properties of ferroelectric materials such as domain wall displacement and conductivity,^8,9^ charged domain walls,^10,11^ tunneling electroresistance phenomena,^12^ electro-optical control of polarization,^13^ and localized photovoltaic effect.^14,15^

Critically, the PFM technique gives direct insight into the local piezoelectric effect and the polarization direction of the ferroelectric domains through amplitude and phase response.^16^ However, the phase and amplitude responses of the PFM signal can be misleading if the signals are not properly calibrated, especially for materials with unknown piezoelectric coefficients. It has been widely reported that artifacts in the PFM signal can originate from localized and nonlocalized electrostatic interactions between the tip–sample and cantilever–sample, ion migration, Joule heating from leaky samples, complex cantilever dynamics, extrinsic contributions, and so on.^17−23^ Hence, uncalibrated PFM signals could create ambiguity associated with different reported results on a similar material owing to the influence of nonintrinsic and external contributions. In contrast, a properly calibrated PFM response can probe intriguing phenomena at the nanoscale, such as the negative piezoelectric coefficient, antiferroelectric phase transition, and coexistence of positive and negative piezoelectric coefficient in the same system.^18,24,25^ For example, materials with positive and negative piezoelectric coefficients can be identified through the clockwise and anticlockwise rotation of the phase loop.^18^ Also, the antiferroelectric to ferroelectric phase transition has been established from amplitude loops exhibiting four minima, whereas typical ferroelectrics display only two minima in the amplitude loop.^24^ However, in order to probe such nanoscopic phenomena, the nonintrinsic contribution of the PFM signal needs to be separated from its intrinsic response. In this context, Balke et al. described the effect of the cantilever stiffness on the rotation of the PFM phase loop.^16^ Recently, Buragohain et al. also demonstrated the importance of the phase offset on the rotation of the phase loops.^18^ It is established that factors such as film thickness, electrode materials, and deposition method can affect the rotation of phase loops in hafnia-based ferroelectric systems, which is linked to the sign of the piezoelectric coefficient and is extremely influenced by the calibrated phase offset. H. Tan et al. demonstrated a clear PFM phase contrast with a 180° phase difference between the up- and down-oriented domains obtained by applying a compensation voltage to the ferroelectric surface.^26^ The selection of the polarity of this compensation voltage was determined by the nature of the *I*–*V* response of the samples. However, the effect of the compensation voltage on the phase and amplitude loops was not demonstrated in their work. Also, the crosstalk between PFM response and other material functionalities such as elastic modulus and cantilever dynamics was demonstrated to be removed from the real ferroelectric response using an interferometric displacement sensor (IDS) with AFM techniques. The use of an IDS with the AFM technique was demonstrated to enable the decoupling of unwanted cantilever motion from tip displacements.^17,27^ Although various calibration processes have been described to obtain accurate local electromechanical response, the effect of calibration on the PFM phase imaging and its correlation with the local electromechanical response has not been explored.

Additionally, ascertaining the reliability of the spatial variation of calibrated PFM phase and amplitude loops at the nanoscale is a foremost aspect of ferroelectric memory applications.^28^ Apart from fatigue and retention loss, one of the important measures to identify the reliability of ferroelectric memory devices is the testing of the imprint effect of the systems.^28−31^ The imprint effect in a ferroelectric system can be identified by measuring a horizontal shift in the hysteresis loops (*C*–*V*, *P*–*E*, strain*–E*, and PFM phase–amplitude), which is caused by the preference of one polarization state over another.^28^ This imprint effect is generally observed owing to a few important factors such as the flexoelectric phenomenon, formation of defect dipoles related to oxygen vacancies, formation of nonswitching layers at domain boundaries, and formation of a dead layer near the ferroelectric/electrode interface.^31−34^ The control of imprint of ferroelectric thin films was also demonstrated by functionalizing the top layers, which causes chemically induced surface polarization pinning in the materials.^35^ Also, measuring the system’s local coercive and imprint voltage can aid in determining the mechanical strain, chemical defects, and fatigue behavior and selecting ferroelectric materials that exhibit optically controlled reversible polarization response for developing brain-inspired computing.^32,36−38^ Therefore, accurately determining the imprint effect can convey several important pieces of information about the sample. Although the imprint behavior in ferroelectrics has been widely studied using nanoscopic PFM techniques, the nonintrinsic contributions in the actual PFM signal question the reliability of these results.^28−31,39−44^

In this work, we describe how to obtain artifact-free PFM phase and amplitude images from ferroelectric materials. The identification of the true PFM response is established from post-PFM phase image analysis. Furthermore, a methodology is developed to minimize the electrostatic contribution in PFM phase–amplitude loops by applying an additional voltage to the tip, which is the same as the potential of the top surface identified by KPFM. Using this developed technique, we then determine the correct offset voltage to minimize the electrostatic potential on the sample surface. Additionally, the obtained methodology is implemented to determine the local imprint voltage by performing the statistical average of the calibrated phase and amplitude loops in polycrystalline BiFeO_3_ (BFO), single-crystal BaTiO_3_ (BTO), epitaxial BTO, and vertically aligned BTO:Sm_2_O_3_ (BTO:SmO) and BTO:MgO epitaxial nanocomposites. Overall, we explore how properly calibrated PFM phase and amplitude signals can provide viable information from ferroelectric domains at the nanoscale and differentiate the phase response for ferroelectrics with different structures and geometries.

## 2 Experimental Methods

### Synthesis of BiFeO_3_ (BFO) Thin Films

BFO thin films were fabricated on FTO substrates using a chemical solution deposition method. To prepare the BFO solutions, bismuth(III) nitrate pentahydrate (Bi(NO_3_)_3_·5H_2_O, 98%; 10% excess) and iron(III) nitrate nonahydrate (Fe(NO_3_)_3_·9H_2_O, 98%) were used as precursors. The precursors were dissolved in a mixed solvent of 2-methoxyethanol (anhydrous, 99.8%) and acetic acid (glacial, ≥99.7%) to form a 0.5 M final solution. The final solution was spin-coated on cleaned FTO substrates at 3000 rotations per minute for 30 s followed by drying at 90 °C for 1 min and 350 °C for 5 min on a hot plate. Finally, films were annealed at 650 °C for 1 h in a tube furnace in an ambient atmosphere.

### Synthesis of BTO, BTO:SmO, and BTO:MgO Thin Films

Epitaxial BaTiO_3_, BTO:Sm_2_O_3_ (denoted as BTO:SmO), and BTO:MgO were fabricated on Nb:STO(001) substrates by pulsed laser deposition. BTO:SmO and BTO:MgO targets were prepared by a solid-state synthesis route using 1:1 BTO and Sm_2_O_3_ powder and 2:1 BTO and MgO powder, respectively, as a precursor purchased from Sigma-Aldrich. BTO and BTO:SmO films were deposited at 800 °C at 150 mTorr of oxygen partial pressure (PO_2_). The films were deposited by ablating the target by using an excimer laser (3 Hz pulse frequency and 2.1 J/cm^2^ fluence) for 20 min. BTO:MgO film was deposited at 835 °C for 50 min and 0.5 Hz. The thickness of all films synthesized by the PLD technique is ∼100 nm.

### PFM and KPFM Measurements

PFM measurements were carried out using a Bruker Dimension Icon with ScanAsyst AFM (Nanoscope-6) system in contact resonance mode.^16,45−47^ All PFM measurements were performed using platinum- and iridium-coated tips (SCM-PIT-V2, Bruker) with a force constant of ∼3 N/m. Prior to the measurements, probes were calibrated by accurately determining the cantilever sensitivity (∼68 nm/V) and the cantilever spring constant (2.9 N/m) from the force versus distance curve and thermal tuning technique, respectively. PFM measurements were performed by applying the bias voltage to the probe, and the bottom electrode was grounded. The phase and amplitude responses were extracted in SS-PFM mode. The SS-PFM mode involves the application of a series of write voltage (“on-field”) segments for each of several read voltages (“off-field”). The SS-PFM data were collected with minimum and maximum writing voltage segments ranging from −10 to +10 V, respectively, in 50 steps. The hold time for the measurement is 50 ms. All PFM phase and amplitude responses were extracted in the “off-field” segments by using a Python script. Data-Cube PFM was measured at the contact resonance frequency by sweeping the forward and backward sweep voltages from −10 to +10 V. The hold time for the measurements was 15 ms. Also, during the SS-PFM measurements, the force between the tip and sample surface is kept constant to avoid a change in the contact resonance frequency and cantilever sensitivity value. It is to be noted that the selected measurements in our work were carried out only on out-of-plane domains; therefore, crosstalk between the in-plane and out-of-plane domain configurations on the SS-PFM measurements is avoided in these systems. The angle-resolved PFM response for BFO and BTO samples confirms that there is no in-plane domain present in the system, as shown in Figures S1 and S2. KPFM measurements were performed using the same probe used in the PFM measurements. The KPFM measurements were executed by keeping the lift distance set to zero.^38^

## 3 Results and Discussion

**Figure 1:**
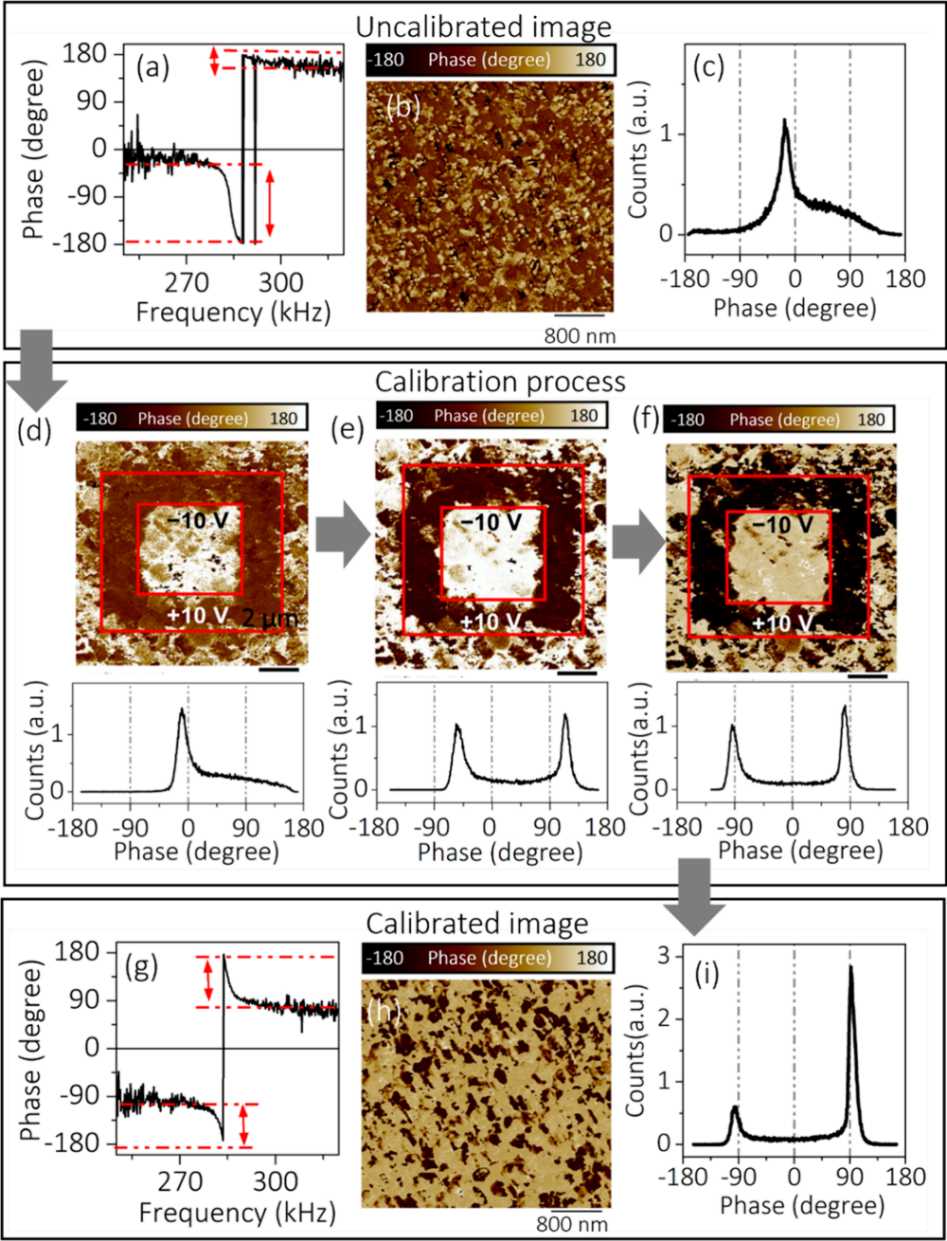
Phase sweep with respect to frequency, PFM phase image, and domain distribution of the BFO thin film measured under (a–c) uncalibrated conditions. After electrical writing, PFM phase images and domain distribution of a BFO thin film in (d) uncalibrated, (e) partially calibrated (autocalibrated), and (f) fully calibrated conditions. Images were taken in 6 × 6 μm^2^ after ±10 V poling in the 4 × 4 and 2 × 2 μm^2^ areas, respectively. Phase sweep with respect to frequency, PFM phase image, and domain distribution of the BFO thin film measured under (g–i) calibrated conditions.

Irrespective of standard good PFM measuring practices in terms of cantilever sensitivity, spring constant, and position of the laser on the cantilever, it is still fully possible for phase offset to affect the orientation of the domain patterns.^16,18,20,39^ In this context, we captured PFM images on polycrystalline BFO thin films to distinguish between artifacts and true PFM images. A polycrystalline BFO thin film was selected to demonstrate the PFM calibration procedure owing to its many crystal orientations.^48−50^ To see the difference between PFM phase images recorded under uncalibrated and calibrated conditions, PFM images were recorded at the contact resonance frequency with either zero phase offset (uncalibrated) or adjusted phase offset (calibrated). The phase sweep with respect to frequency for the uncalibrated condition is plotted in Figure 1(a). The amplitude response of the sample fits with the simple harmonic oscillator (SHO) function and reveals a symmetric fitting with the coefficient of determination (*R*^2^) value close to 1, regardless of phase offset calibration.^16^ The topography of the film and respective plots are displayed in Figure S3. It is important to understand that even though the measurement was taken under uncalibrated conditions, the amplitude response’s SHO fitting displayed a symmetric behavior. Additionally, the phase response at the resonance frequency exhibited the characteristics of a calibrated electromechanical response: a difference of 180°.^16^ However, it is noticed that the shape of the phase sweep is asymmetric with a different peak-to-plateau offset before and after the contact resonance frequency, as highlighted in Figure 1(a). The non-180° phase difference at resonance and asymmetric phase offset has been considered as originating from cantilever dynamics, sampling delay, and even from the cable used in the instrument.^16,18^ To correlate the uncalibrated phase sweep with the PFM imaging, the obtained PFM phase image and corresponding domain distribution of the samples are shown in Figure 1(b) and (c). The bright and dark regions in the PFM phase image correspond to up (positive phase difference) and down (negative phase difference) oriented domains, respectively. In the uncalibrated condition, the domain distribution illustrates that most of the domains in the sample appear to be oriented in one direction. Moreover, the phase image does not clearly illustrate two different types of domains present in the sample.

A good way to check the correct phase offset is to pole a region, which should result in only ‘up’ and ‘down’ domains. Therefore, to obtain the correct phase offset in the system, domains in the BFO thin film were aligned in the upward and downward directions by poling with −10 and +10 V, which will place the phase difference in the respective areas at +90° and −90°. We poled the BFO thin film with +10 and −10 V on 4 × 4 and 2 × 2 μm^2^ areas. Later the whole 6 × 6 μm^2^ region was scanned with a 2 V drive amplitude in three phase-offset conditions (uncalibrated, partially calibrated, and fully calibrated). The PFM phase images are obtained in uncalibrated (phase offset = 0°), partially calibrated, using auto phase offset in the software (phase offset = −32°), and fully calibrated (phase offset = −66°) conditions. The respective images are depicted in Figure 1. Figure 1(d) depicts that the phase offset is incorrect, as domains are not perfectly aligned to the field directions. After performing the auto-phase offset through the inbuilt software, the instrument itself does an initial calibration for the phase offset. Figure 1(e) is recorded after auto-phase calibration. It is observed that the contrast of the PFM phase image in the same area is improved significantly after auto-phase calibration and so is the domain distribution. Although the phase difference between the up- and down-oriented domains is found to be ∼172°, the up- and down-oriented domains are positioned at −56° and +116°, respectively, as shown in Figure 1(e). Afterward, manual calibration was applied by incorporating an additional drive phase of −34° to the phase offset achieved after the autocalibration. This process aligns the down- and up-oriented domains at approximately −90° and +90°, respectively. The phase image after manual calibration is depicted in Figure 1(f). It is to be noted that after the calibration, the slight deviation of the phase value for down- and up-oriented domains from −90° and +90°, respectively (Figure 1f) could be caused by the interaction between the tip and local surface charge.^26^

After the correct phase offset was obtained (calibrated), the phase sweep with respect to frequency was acquired for the BFO sample and is plotted in Figure 1(g). When a calibrated phase offset condition is obtained, a symmetric phase response can be observed while the amplitude response with frequency remains consistent. Here, in the phase sweep curve, the plateaus before and after the contact resonance frequency are (anti)symmetric. The corresponding PFM image and domain distribution of the samples are shown in Figure 1(h). It should be noted that the initial uncalibrated (Figure 1b) and final calibrated (Figure 1h) PFM image measurements were performed at the same position, where poling and calibration (Figure 1d) were performed on an adjacent region, as shown in Figure S4. After the calibration procedure, the domain distribution of the sample reveals that both up- and down-oriented domain distributions are represented in the BFO system with a 180° phase difference between them. Therefore, the correct phase offset is important not only in obtaining the calibrated phase loop but also in obtaining the domain distribution from the PFM phase image. It is concluded that prior to PFM image capture, full phase offset calibration is mandatory to obtain the correct information about the domain configuration of the system.

**Figure 2:**
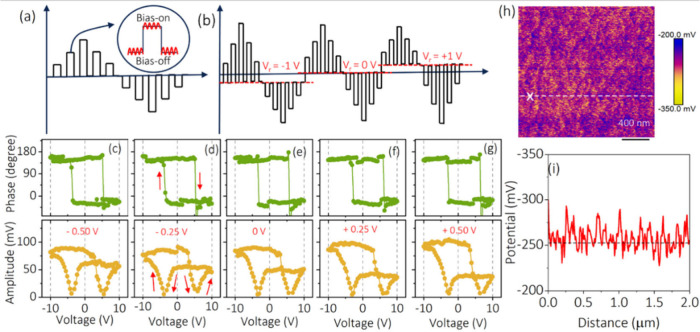
(a) The schematic of single-segment SS-PFM script showing the voltage waveform in bias on and off conditions. (b) Example multisegment SS-PFM script displaying write and three read voltage steps, where read voltage steps are −1, 0, and +1 V. (c −g) PFM phase and amplitude hysteresis loop of a BFO thin film measured for −0.50 to +0.50 V read voltages, respectively. The electrostatically neutral phase amplitude loop is displayed in part d. (h) KPFM image (2 × 2 μm^2^) and (i) KPFM line scan of the BFO thin film in the measured SS-PFM area.

While a calibrated PFM phase image can provide useful insights into domain morphology and evolution, the SS-PFM technique is a valuable tool to obtain detailed local piezoelectric responses.^23,51^ Importantly, SS-PFM allows electrostatic-free phase and amplitude loops measured under the bias-off (off-field) conditions to be obtained. In a typical SS-PFM measurement, *V*_Tip_ = *V*_probe_(*t*) + *V*_AC_ cos ω*t* is applied to the tip, where *V*_AC_ is the driving amplitude. The voltage waveform applied in the SS-PFM technique is schematically shown in Figure 2(a). However, the conventional input voltage script displayed in Figure 2(a) with a zero read voltage cannot completely account for the local electrostatic interactions. In a typical PFM setup, the electrostatic force *F*_elect_ is proportional to the capacitance gradient ∂*C*/∂*z* and the square of the potential difference between the tip (*V*_Tip_) and sample surface (*V*_S_), which can be expressed as *F*_elect_ = 1/2 ∂*C*/∂*z*(*V*_Tip_ – *V*_S_)^2^. The contribution of the capacitance gradient consists of local  and nonlocal capacitance gradients  originating from tip–sample and cantilever–sample interactions, respectively. Finally, the first harmonic of the piezoelectric signal PR_1ω_ can be expressed as^18^
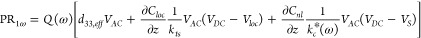
1where ω is the frequency of the AC voltage, *Q*(ω) is a frequency-dependent scaling factor originating from the cantilever dynamics, *V*_loc_ and *V*_S_ are the local and averaged surface potential, respectively, *k*_ts_ is the effective tip–sample contact stiffness, and *k*_c_^*^(ω) is the frequency-dependent effective cantilever stiffness. Therefore, eq 1 illustrates that both the local and averaged sample surface potentials can influence the piezoelectric response. Therefore, the SS-PFM technique was updated to balance the surface charge effect of the sample by incorporating different read voltage (*V*_r_) segments, as shown in Figure 2(b).^20,51,52^Figure 2(b) depicts the schematic of a SS-PFM voltage wavefront having −1, 0, and +1 V *V*_r_ segments. However, the selection of the electrostatically neutral phase amplitude loops measured under different *V*_r_ values is poorly described in the literature. In this context, we describe a new methodology to accurately select electrostatically neutral phase amplitude loops obtained from ferroelectric samples.

The condition for obtaining electrostatically neutral phase–amplitude loops is when *V*_r_ is equal to the surface potential of the sample. In order to explore a more rigorous method to obtain electrostatically neutral PFM phase amplitude loops, we measured the PFM phase and amplitude loops on a polycrystalline BFO thin film. Prior to that, we followed a standard practice of calibration on the BFO thin film, as described in the previous section. In the SS-PFM measurements, the BFO thin film was subjected to a voltage signal involving a series of write voltage segments for each of several (“off-field”) *V*_r_. The SS-PFM data were collected with minimum and maximum write voltage segments ranging from −10 and +10 V, respectively, in 50 steps. The write time duration of each segment was 10 ms. The minimum and maximum *V*_r_ segments ranged from −1 to +1 V, in 9 steps, going from low to high. The waveform of the applied signal is displayed in Figure S5. The obtained phase and amplitude loops against bias voltage for a BFO thin film are plotted in Figure 2 at *V*_r_ = −0.50, −0.25, 0, +0.25, and +0.50 V. The obtained phase and amplitude loops at *V*_r_ = −1, −0.75, +0.75, and +1 V are plotted in Figure S6. In Figure 2, the phase and amplitude loops at the respective read voltages illustrate typical ferroelectric behavior with increasingly asymmetric amplitude response. Also, the phase and amplitude loops for the BFO sample were found to shift increasingly toward the right side as the read voltage was varied from *V*_r_ = −0.50, −0.25, 0, +0.25, and +0.50 V. As typical phase and amplitude curves are obtained for a BFO thin film at all *V*_r_, a question arises: which read voltage provides the correct phase and amplitude response of the samples? From eq 1, it is noticeable that the surface potentials (*V*_S_ and *V*_loc_) and *V*_DC_ (*V*_r_) should be equivalent in magnitude to balance the electrostatic force on the sample. In this context, the surface potential of the BFO is measured using KPFM and is displayed in Figure 2(h). The line profile of the surface potential image near the measured point illustrates the value of −0.253 V. Therefore, the phase–amplitude curve corresponding to *V*_r_ = −0.25 V can be considered as the electrostatically neutral response and therefore provides the correct information about the samples. It is to be noted that to verify the probe itself is actively not modifying the surface potential during SS-PFM measurements, KPFM measurements were carried out on the BFO thin film before and after SS-PFM measurements. We observed that KPFM surface potential does not change as a result of the AFM probe’s interaction with the surface. The KPFM image of the BFO thin film before and after SS-PFM measurements is displayed in Figure S7.

**Figure 3:**
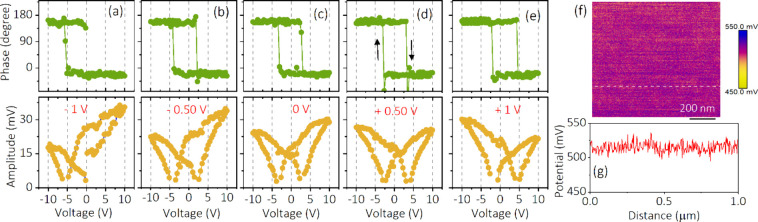
(a–e) PFM phase and amplitude hysteresis loop of a BTO single crystal for −1 to +1 V read voltage segments. (f) KPFM image (1 × 1 μm^2^) and (g) KPFM line scan in the measured SS-PFM area.

To validate this methodology in a different type of ferroelectric sample, the phase and amplitude curves were also measured in a commercially procured (MTI Corporation) one-side-polished BTO single crystal.^53^ The SS-PFM measurements on the BTO crystal were performed in a region with out-of-plane domains. Prior to the measurements, for good calibration practices the SHO fittings of amplitude, phase sweep, and *R*^2^ value for the BTO crystal were obtained, which are displayed in Figure S8, in this case, irrespective of the fact that typical ferroelectric phase and amplitude curves are observed at different *V*_r_. It is noticeable that the phase and amplitude loops for the BTO sample are found to prominently shift from the left to right side as the *V*_r_ is increased, as shown in Figure 3. Phase and amplitude loops at *V*_r_ = −1, −0.50, 0, +0.50, and +1 V illustrate typical ferroelectric behavior with a varying asymmetry in the amplitude response. To obtain electrostatically neutral phase amplitude loops, the loop corresponding to *V*_r_ = +0.50 V is selected as a surface potential of +0.51 ± 0.07 V was obtained near the measured point of the BTO crystal (Figure 3f). It is noticed that at *V*_r_ = +0.50 V BTO exhibits a symmetric amplitude response. Such a symmetric amplitude response has previously been observed,^54^ which therefore validates our approach. However, considering the case of BFO polycrystalline films in Figure 2, it can be seen that such a symmetric response is not always obtained, thus demonstrating the benefit of using KPFM to provide a quantitative measure of the required electrostatic offset. We have summarized a few important factors that influence the PFM response and present them in Table S1. Overall, the established methodology describes a process to select electrostatically neutral phase amplitude loops in the ferroelectric system by combining SS-PFM and KPFM techniques, which eventually helps to determine the true response of local ferroelectric characteristics at the nanoscale. Note that in order to eliminate the long-range electrostatic force influencing the electromechanical response in the BFO thin film and BTO crystal, the amplitude response is captured by sweeping the tip bias 100 nm away from the surface, as shown in Figure S9. The noise-like amplitude versus voltage response illustrates that there is no long-range force influencing the overall electromechanical response in these systems.^17^ Additionally, the comparison of phase loops for calibrated and uncalibrated conditions is shown in Figure S10 for the BFO thin film and Figure S11 for the BTO crystal.

**Figure 4:**
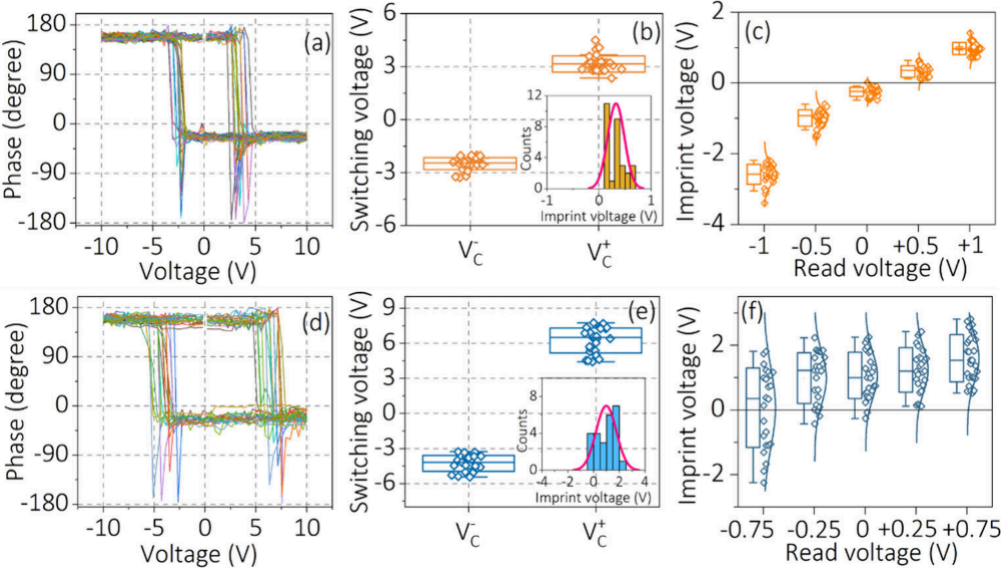
(a, d) Phase versus bias voltage and (b, e) switching voltage measured at the correct *V*_r_ value obtained from several measurements for a BTO single crystal and BFO thin film, respectively. (c and f) Variation of imprint voltage obtained under different *V*_r_ values.

By developing a quantitative methodology for determining the correct read voltage to offset any electrostatic effects in SS-PFM, we are now able to implement this approach to accurately determine the local switching and imprint voltage of ferroelectric BTO and BFO systems. Notably, the imprint voltage of the systems can be obtained by *V*_imp_ = (*V*_C+_ + *V*_C–_)/2, where *V*_C+_ and *V*_C–_ represent the positive and negative coercive voltage, respectively,^27,55^ but these can only be accurately obtained if electrostatic offsets have been correctly accounted for. Therefore, we determined that *V*_r_ = +0.5 V gives the electrostatically neutral response. In Figure 4(a), we summarize the phase loops measured at 30 different points of the BTO crystal at *V*_r_ = +0.5 V to determine the imprint effect in the system. Generally, the difference in magnitude of *V*_C+_ and *V*_C–_ confirms the existence of imprint character in the materials. It is observed that the phase loop of the sample is shifted toward the right (*V*_imp_ > 0), illustrating that an imprint field exists (*E*_imp_ > 0). The switching voltage was extracted from the phase loops and is plotted in Figure 4(b). The positive imprint voltage (*V*_C+_ > *V*_C–_) observed in the BTO sample is also displayed in the histogram of the inset of Figure 4(b). To understand the importance of selecting the correct *V*_r_ value, the perceived *V*_imp_ of the BTO sample extracted from different *V*_r_ segments is displayed in Figure 4(c). Note that *V*_imp_ is extracted from the phase loops measured at different *V*_r_. Figure 4(c) reveals that the perceived *V*_imp_ of the sample shifted from negative to positive with different *V*_r_. For example, the *V*_imp_ obtained from the phase loops measured at *V*_r_ = 0 V illustrates a shift toward the negative side (*V*_imp_ < 0). However, *V*_imp_ = +0.35 V obtained from the phase loops measured at *V*_r_ = +0.5 V—equivalent to surface potential—illustrates a shift toward the positive side (*V*_imp_ > 0). This clearly demonstrates that it is essential to determine the correct *V*_r_ to get accurate information about the nanoscopic electromechanical properties of the sample, as each *V*_r_ value gives a different perceived *V*_imp_, and only by determining the correct *V*_r_ can the correct *V*_imp_ be obtained. It is also to be noted that the influence of the local charging effect on the imprint value obtained in our work cannot be completely ruled out, which might arise due to the application of the voltage to the probe during the measurements.

Similarly, phase and amplitude loops are also measured at *V*_r_ = −0.25 V in 30 different points on the polycrystalline BFO thin film to ascertain the *V*_imp_ of the sample. The measured phase loops are displayed in Figure 4(d). The average imprint voltage (+1.21 V) ascertained from the histogram of the phase loops in the polycrystalline BFO thin film is found to be shifted to the positive side (*V*_imp_ > 0), as shown in the inset of Figure 4(e). There is also a slight trend in the *V*_imp_ distribution with different *V*_r_ values (Figure 4f), similar to the BTO single-crystal sample. However, this is small in comparison to the large standard deviation within each *V*_r_. Overall, this signifies that the correct *V*_imp_ of any ferroelectric sample can be determined from the calibrated phase–amplitude loops by determining the correct read voltage from the KPFM measurements. On the other hand, using the incorrect read voltage leads to the incorrect *V*_imp_ value and therefore misinterpretation of the overall outcomes. Note that during the loop measurements in BFO and BTO systems, we have kept the force between the tip and surface constant to avoid complex cantilever dynamics. For reference, the force, voltage, amplitude, and phase versus measurement time at two different pixels are shown in Figures S12 and S13 for a BFO thin film and BTO crystal, respectively.

**Figure 5:**
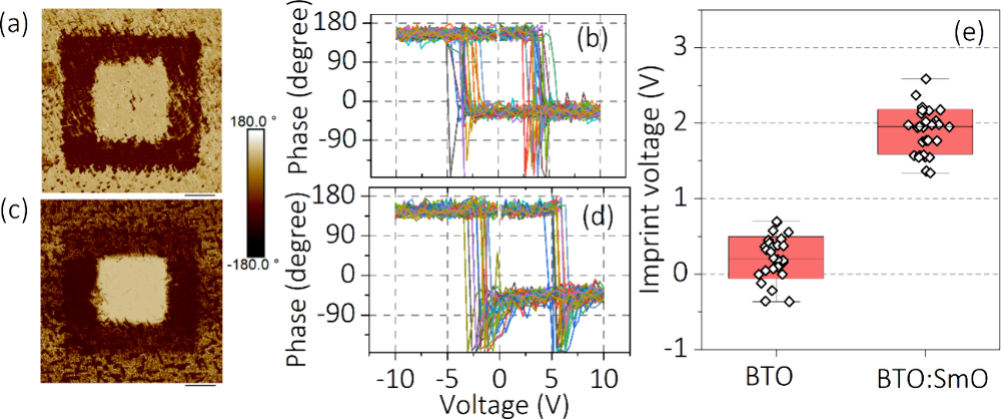
PFM phase response exhibiting the ferroelectric domain switching behavior and phase loops of (a and c) BTO and (b and d) BTO:SmO VAN structures. Images were taken in 6 × 6 μm^2^ after ±10 V poling in the 4 × 4 and 2 × 2 um^2^ areas, respectively. (e) The imprint voltages for BTO and BTO:SmO VAN structures.

To illustrate the versatility of the newly developed methodology, the imprint voltage is determined in different strain states of epitaxial BTO thin films. Thus, this methodology is implemented on a classic ferroelectric BTO epitaxial film and a BTO:SmO vertically aligned nanocomposite (VAN) grown on Nb:STO(001) substrates.^56^ The surface morphologies of BTO and BTO:SmO VAN structures are displayed in Figure S14. The VAN microstructure consists of two epitaxial phases separated by vertical interfaces. The lattice mismatch generates an out-of-plane strain along the thickness exerted by the stiffer phase onto the softer phase. Note that the BTO:SmO VAN structure creates out-of-plane 2.3% tensile strain in BTO.^56^ First, calibrated PFM phase images are acquired after applying ±10 V on the bare surface of the BTO film and BTO:SmO VAN, as shown in Figures 5(a) and (b). In both figures, the brighter/darker regions correspond to regions written with −10/+10 V and read with a 1 V drive amplitude. Both samples exhibit typical ferroelectric-type domain switching. The phase loops are measured in more than 30 cycles on the BTO film and BTO:SmO VAN samples in order to study their imprint characteristics. The respective plots are displayed in Figures 5(c) and (d). To quantify the imprint effect in these systems, the extracted imprint voltages for BTO and BTO:SmO VAN are plotted in Figure 5(c) using calibrated *V*_r_ = +0.50 V obtained from the KPFM surface potential, revealing a positive imprint voltage for BTO:SmO VAN, as compared to the BTO film, which displays minimal imprint voltage. The surface potentials of BTO and BTO:SmO are displayed in Figure S15. From earlier work, as the strain of BTO in the BTO:SmO nanocomposite is controlled by SmO, not by the substrate, the out-of-plane strain relaxation of the VAN film is insignificant compared to the pure BTO thin film.^52^ Therefore, this strain (*u*) generates a huge internal electric field () due to the flexoelectric effect, where *e* is the electronic charge, ε is the permittivity of free space, *a* is in-plane lattice parameter, and  is the strain gradient.^57^ Hence, the observed imprint effect in the BTO:SmO VAN could be associated with the tensile strain-gradient-induced flexoelectric effect in the system, which creates a preferred polarization state in the system.

**Figure 6:**
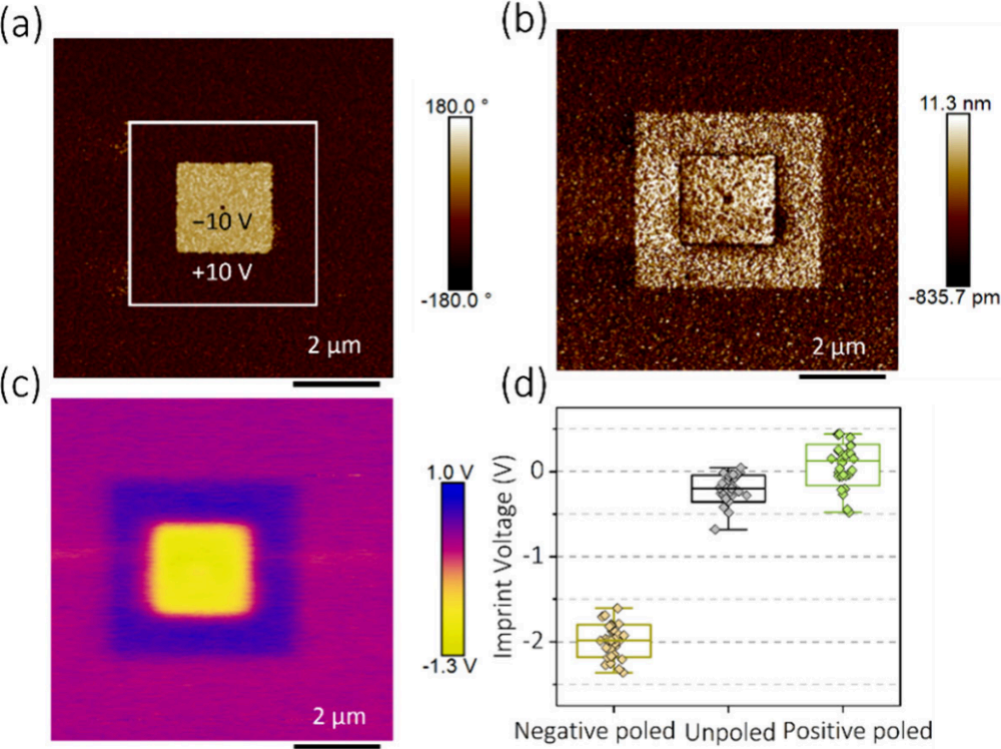
(a, b, and c) PFM phase, amplitude, and KPFM potential of a BTO:MgO VAN structure of an 8 × 8 um^2^ area after ±10 V poling in the 4 × 4 and 2 × 2 um^2^ areas, respectively. (d) Imprint voltage extracted from the sample at the unpoled, positive, and negative poled conditions using *V*_r_ values obtained locally within each region as shown in part (c).

One question that arises from the use of this methodology is how the polarization state of the ferroelectric sample itself affects the surface potential and therefore the perceived imprint effect. To explore this effect, we investigate the electric field-induced imprint behavior of a BTO:MgO VAN structure. In the case of BTO:MgO VAN films, poling of the sample using the PFM probe demonstrates that the domain in the BTO:MgO VAN is initially oriented in a downward direction, exhibiting darker contrast in the PFM phase image, which is unchanged with the application of positive voltage (+10 V) (Figure 6a). On the other hand, with the application of negative voltage (−10 V), the downward domain is completely oriented in the upward direction, resulting in brighter contrast in the PFM phase image. The respective PFM phase and amplitude images are shown in Figures 6(a) and (b). Now, when studied with KPFM, the surface potential is observed to exhibit different values (+0.25, + 0.5, and −1 V) in the unpoled, positive, and negative poled regions, as displayed in Figure 6(c). The phase–amplitude loops are then measured in the respective regions at different *V*_r_ values using the surface potentials obtained from KPFM. The imprint voltage in the respective spots is extracted from the phase loops and is plotted in Figure 6(d). This indicates that the negative poled area exhibits a negative imprint voltage. On the other hand, as the domain orientation in the unpoled and positive poled area is similar, minimal variation of imprint voltage is observed, both showing close to zero imprint voltage. In contrast, the imprint voltages extracted from the phase loops in the unpoled, positive, and negative poled regions at *V*_r_ = 0 V are plotted in Figure S16. In this case, all areas exhibit a negative imprint effect with a slight trend of negative poled < unpoled < positive poled, thus showing very different results compared to the data shown in Figure 6. This demonstrates that the method for obtaining a calibrated PFM phase response demonstrated here can help to differentiate the imprint behavior of a particular ferroelectric domain using the nanoscopic measurements. Note that this indicates that nanoscale variations in surface potential (such as between domains) require the use of different *V*_r_ values across a surveyed area. It would therefore be ideal to have an SS-PFM script incorporating KPFM measurements, in which the surface potential is measured in each pixel prior to the SS-PFM sweep to account for local variations arising from domain patterns. The obtained surface potential value can then be used to automatically choose as a read voltage and perform the SS-PFM measurements at the particular voltage. In this way, the electrostatic contribution in the phase–amplitude loops will be minimized accurately for each pixel/area.

**Figure 7:**
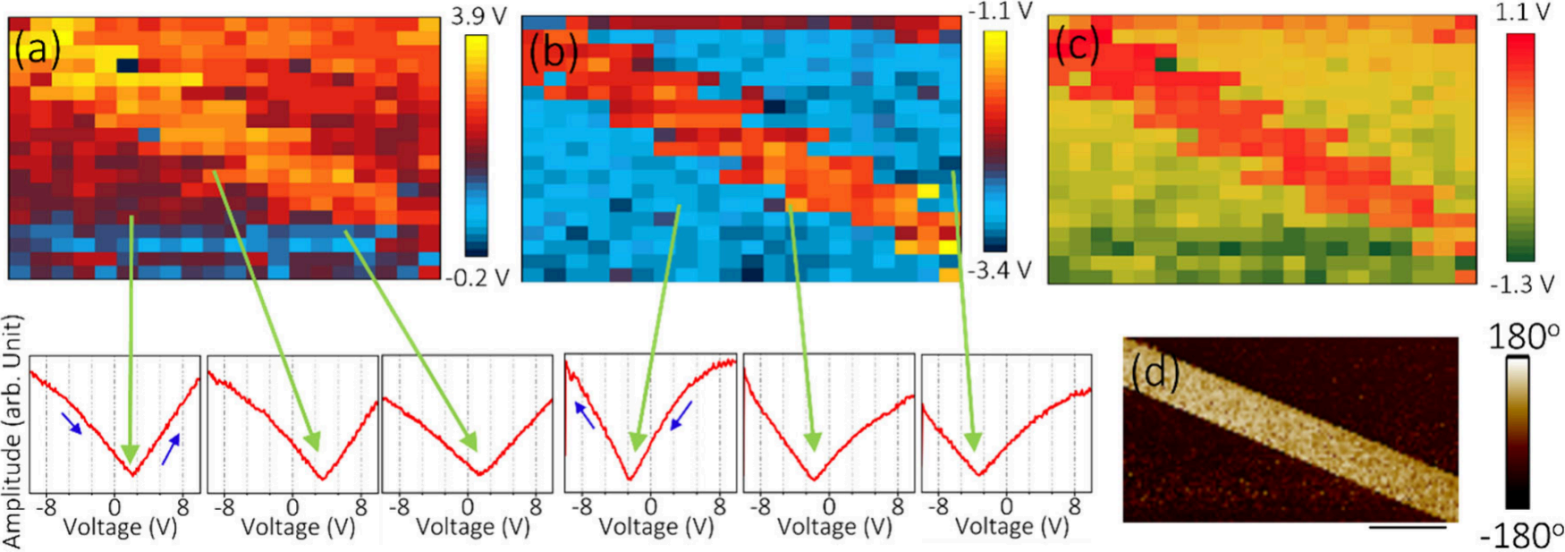
Coercive voltage map acquired from the forward (a) and backward (b) voltage sweeps in a 15 × 10 μm^2^ area, with the respective amplitude sweeps from a selection of pixels shown below, as indicated by the arrows. (c) Imprint voltage map and (d) corresponding PFM phase image of a BTO crystal.

To further illustrate the applicability of SS-PFM to nanoscale characterization, datacube PFM (DCUBE-PFM) is utilized to visualize the 2D distribution map of the switching voltage and imprint voltage over a large area. DCUBE-PFM is an innovative technique to simultaneously perform phase and amplitude loops in each pixel/point from a single data set, providing a detailed picture of the local ferroelectric domains. In this work, DCUBE-PFM is used to visualize the switching and imprint voltage distribution of the BTO single crystal in out-of-plane domains by integrating the developed methodology outlined above. The out-of-plane phase and amplitude images of single-crystal BTO are measured at the calibration condition prior to the DCUBE-PFM measurements. DCUBE-PFM measurements are later performed at the calibrated drive frequency (285 kHz), drive phase (−70°), and DC offset voltage (−0.5 V). The DC offset voltage is applied to the sample at the opposite potential to the top surface to balance the electrostatic interaction. Finally, the phase and amplitude loops are measured at a 15 × 10 μm^2^ area over 20 × 20 pixels. Here the phase and amplitude loops are measured in the forward and reverse voltage sweep, which indicate loops captured during negative to positive and positive to negative voltage sweeps, respectively. Afterward, the point-to-point variations of switching voltage over 15 × 10 μm^2^ were extracted from the amplitude versus voltage loop using Python scripts. The 2D switching voltage map obtained from forward and reverse voltage sweeps is presented in Figures 7(a) and (b), respectively. The corresponding amplitude sweeps are displayed for three pixels each, as examples for clarity. Finally, the 2D map of the imprint voltage is extracted from the switching voltage and plotted in Figure 7(c). It is observed that the up- and down-oriented domains exhibited positive (>+0.5 V) and negative (>−0.4 V) imprint voltage, respectively, and match excellently to the phase image of the same region of the BTO crystal displayed in Figure 7(d). Hence, the 2D imprint voltage in the local ferroelectric domain obtained from the DCUBE-PFM is found to be sensitive to the domain’s orientation, which cannot be concluded from the local SS-PFM measurement. Consequently, the obtained results demonstrate the efficacy of the DCUBE-PFM technique in determining the 2D switching/imprint voltage map in the local ferroelectric domain. To eliminate the fact that the imprint voltage is not influenced by the surface charge artifacts, the surface potential image is captured in the upward and downward domain, as shown in Figure S17. It is also to be noted that although the imprint voltage differs locally depending on the alignment of the domain, the average imprint value in the microscale (15 × 10 μm^2^) exhibits zero imprint value, as shown in Figure S18.

## 4 Conclusion

We have demonstrated a new methodology to accurately obtain the imprint voltage in a range of ferroelectric samples including polycrystalline BFO thin films, BTO single crystals, thin films, and vertically aligned nanocomposite films. In order to achieve this, it was first necessary to undertake detailed calibration to correct parasitic phase offsets using PFM phase switching analysis and adjusting the up- and down-oriented domain to +90° and −90° using established, though often neglected, approaches. Following this, we describe a new technique to quantitatively determine the correct voltage offset to use in SS-PFM measurements in order to minimize electrostatic interactions. This was achieved by measuring phase–amplitude loops in the off-field voltage segment by applying an additional voltage to the tip, which is equivalent to the surface potential of the sample measured using KPFM. We demonstrate that this is more accurate and universally applicable than previous approaches of visually analyzing SS-PFM loops obtained at different offset voltages. By accurately and quantitatively identifying the correct offset voltage, we then were able to determine the imprint voltage in BTO single crystal and polycrystalline BFO thin films.

Interestingly, by applying this technique to a BTO:SmO VAN film, we observe a positive imprint effect compared to a BTO thin film owing to the 2.3% out-of-plane tensile strain in the BTO matrix in BTO:SmO, which produces one preferential polarization over another. In a BTO:MgO VAN thin film the imprint voltage is also determined in the unpoled, positive, and negative poled area, demonstrating a negative imprint voltage corresponding to a negative poled area. This demonstrates both the ability to identify areas of alternate polarization via their imprint but also the need to consider local variations in surface potential when applying this technique. Finally, we have shown that the point-to-point variation of phase/amplitude loops measured using DCUBE-PFM allows the creation of a 2D imprint voltage map of the local ferroelectric domains in a BTO crystal.

Overall, this work highlights how a properly calibrated PFM phase–amplitude signal can provide accurate quantification of local ferroelectric domain switching and imprint effects across a broad range of ferroelectric samples. Furthermore, we demonstrate that this can be extended to produce a 2D switching/imprint voltage map with nanoscale accuracy that is not attainable using macroscopic ferroelectric measurements. This therefore offers a new route to obtain deeper insight in the local ferroelectric properties by minimizing the effects of extrinsic artifacts.

## Data Availability

The data that support the findings of this study are available at Queen Mary Research Online (QMRO) at https://qmro.qmul.ac.uk/xmlui/.

## Supporting Information Available

Angle-resolved PFM of BFO and BTO, topographic image, SHO fitting of amplitude and *R*^2^ value with frequency for BFO; SHO fitting of amplitude, phase sweep with frequency, and *R*^2^ value with frequency for BTO; optical image of a BFO thin film where PFM calibration is performed; SS-PFM waveform; KPFM before and after SS-PFM measurements on BFO; phase image, domain distribution, and phase–amplitude curve of a BFO thin film in the uncalibrated and calibrated conditions; phase–amplitude curve of a BTO thin film measured in uncalibrated and calibrated conditions; off-surface SS-PFM measurements, topographic image of BTO and BTO:SmO thin films; KPFM image of BTO and BTO:SmO; imprint variation at *V*_r_ = 0 for BTO:MgO; force, voltage, amplitude, and phase versus measurement time for BFO and BTO samples (PDF)
Supporting video file demonstrating the calibration procedure of a BFO thin film (MP4)
