# Supplementary material for: Determination of Imprint Effects in Ferroelectrics from the Quantified Phase and Amplitude Response
Source: ACS Appl Electron Mater. 2024 Sep 16;6(9):6401–10. doi: 10.1021/acsaelm.4c00875 (PMC11425849; doi:10.1021/acsaelm.4c00875)
Supplement: Supplementary file 1 — el4c00875_si_001.pdf [file el4c00875_si_001.pdf]

SUPPORTING INFORMATION

Determination of Imprint Effects in Ferroelectrics from the Quantified Phase and Amplitude Response

Subhajit Pal[1], Emanuele Palladino[1], Haozhen Yuan[1], Muireann Anna de h-Óra[2], Judith L. MacManus-Driscoll[2], Jorge Ontaneda[1], Vivek Dwij[3], Vasant G. Sathe[3], and Joe Briscoe[1*]

[1]School of Engineering & Materials Science, Queen Mary University of London, London E1 4NS, United Kingdom

[2]Department of Materials Science & Metallurgy, University of Cambridge, 27 Charles Babbage Road, Cambridge CB3 0FS, United Kingdom

[3]UGC-DAE Consortium for Scientific Research, University Campus, Khandwa Road, Indore 452017, India

[*]Corresponding author: *j.briscoe@qmul.ac.uk*

Contents

1. Angle-resolved PFM for BFO and BTO samples.
2. PFM calibration details of BFO and BTO samples.
3. Optical image of BFO thin film where the calibration is performed.
4. Voltage waveform applied for the SS-PFM measurements.
5. KPFM before and after SS-PFM measurements for BFO thin films.
6. Table summarising of most influential factors which manipulate the true PFM results and their possible solutions.
7. Off-surface SS-PFM measurements of BFO and BTO samples.
8. Force, voltage, amplitude and phase versus measurement time for BFO thin film and BTO crystal at two different pixels.
9. Topography and KPFM measurements of epitaxial BTO and BTO:Sm_2O_3 thin films.
10. Imprint voltage of BTO:MgO sample in the unpoled, positive, and negative poled conditions at Vr = 0 V.
11. Topography, PFM and KPFM of BTO crystal at the same place.
12. Imprint voltage average distribution and 2D voltage map of the BTO crystal.

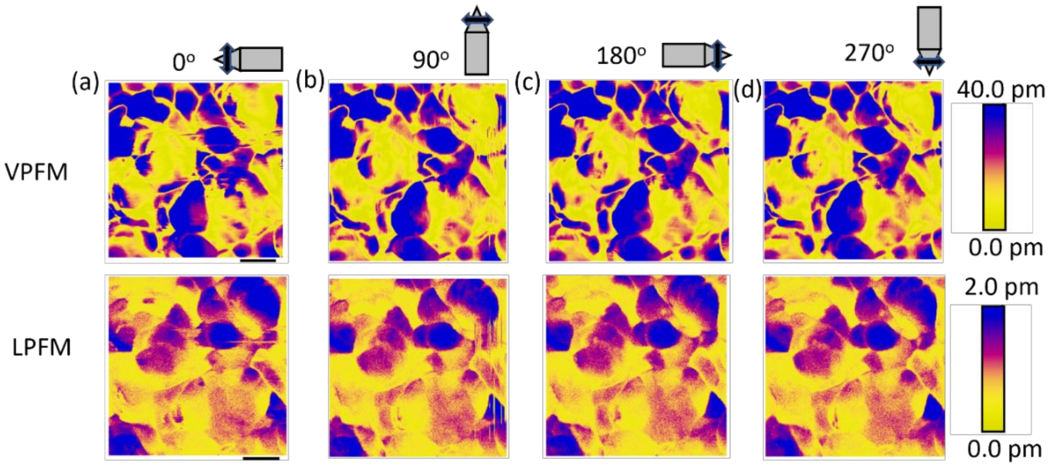

Figure S1. Out-of-plane (VPFM) and in-plane (LPFM) amplitude response of BFO thin film with (a) 0°, (b) 90°, (c) 180° and (d) 270° scan angles.

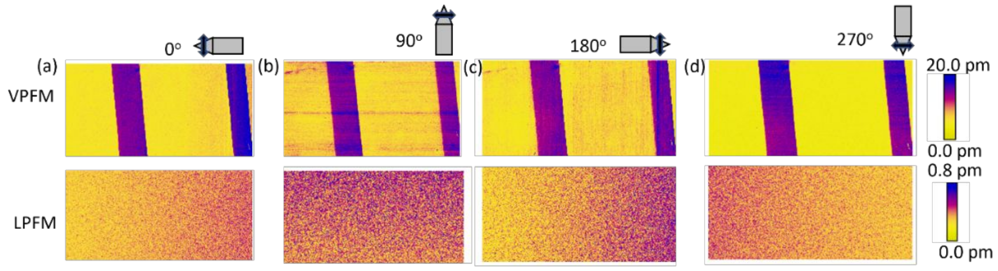

Figure S2. Out-of-plane (VPFM) and in-plane (LPFM) amplitude response of BTO crystal with (a) 0°, (b) 90°, (c) 180° and (d) 270° scan angles.

Angle-resolved PFM measurements were performed by keeping the sample fixed and rotating the scan angle during the measurements. It is observed that the in-plane amplitude response (LPFM) does not change with the scanning angle in both BFO and BTO samples, which illustrates there is no in-plane domain present in these systems. The out-of-plane amplitude (VPFM) response is added for the reference.

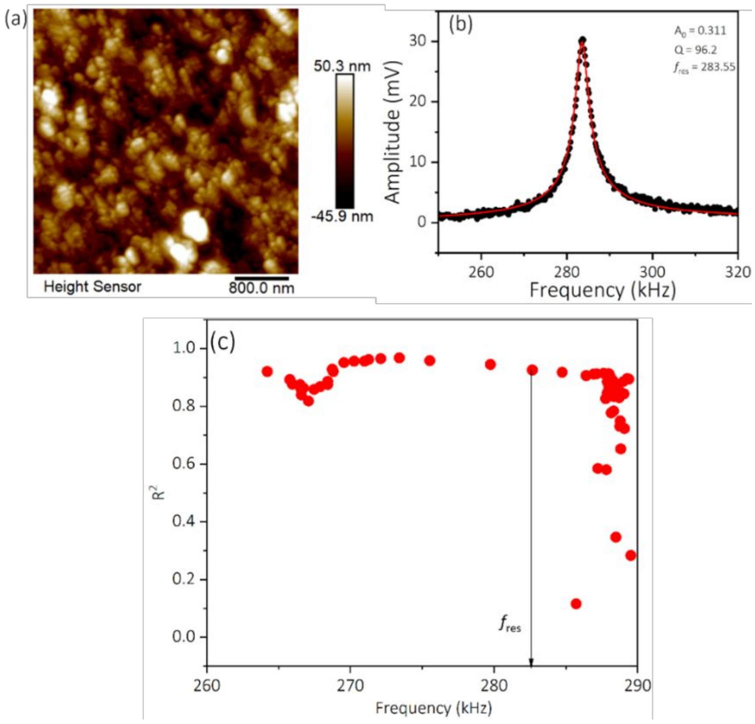

Figure S3: (a) Topography image of BFO thin film. (b) Amplitude fitting across the contact resonance frequency. (c) R^2 value with frequency for BFO.

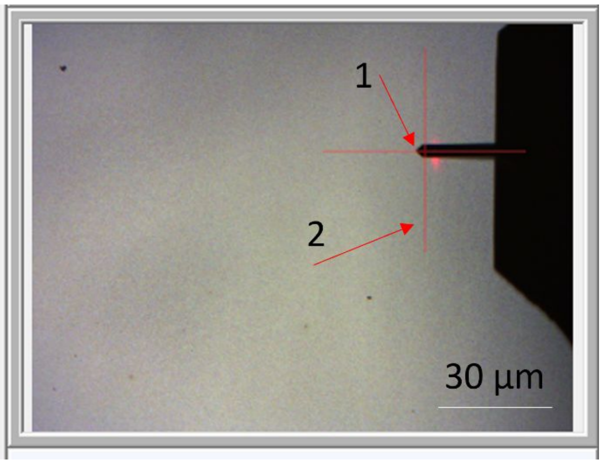

Figure S4: Optical image of BFO thin film where PFM calibration is performed. Place-1; where uncalibrated (Fig. 1 (a), (b), (c)) and calibrated (Fig. 1 (g-i)) images are taken. Place 2, where the calibration process (Fig. 1 (d-f)) is carried out.

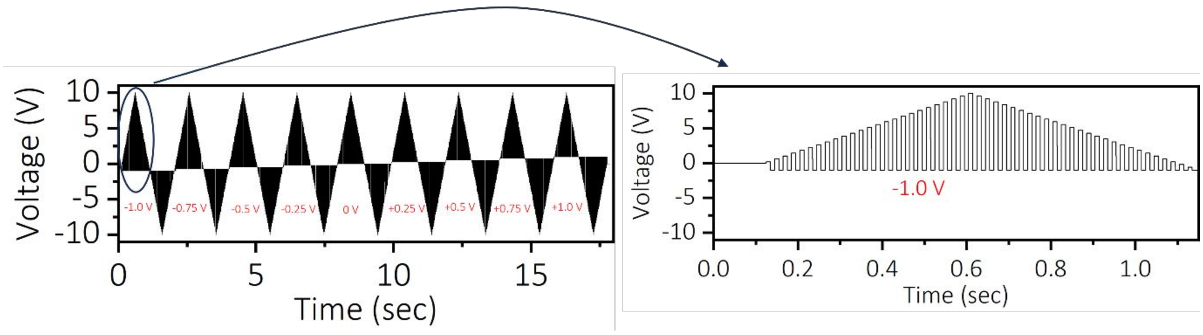

Figure S5: Voltage waveform applied for the SS-PFM measurements.

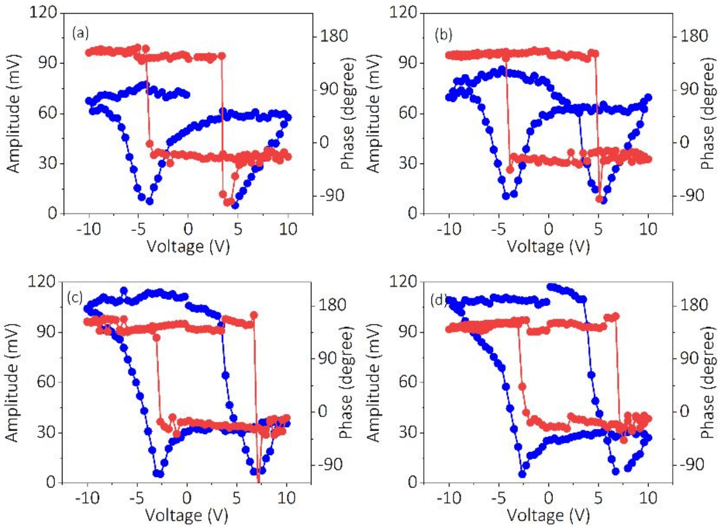

Figure S6: PFM phase and amplitude hysteresis loop of BFO thin film measured at Vr = (a) −1, (b) −0.75, (c) +0.75, and (d) +1 V.

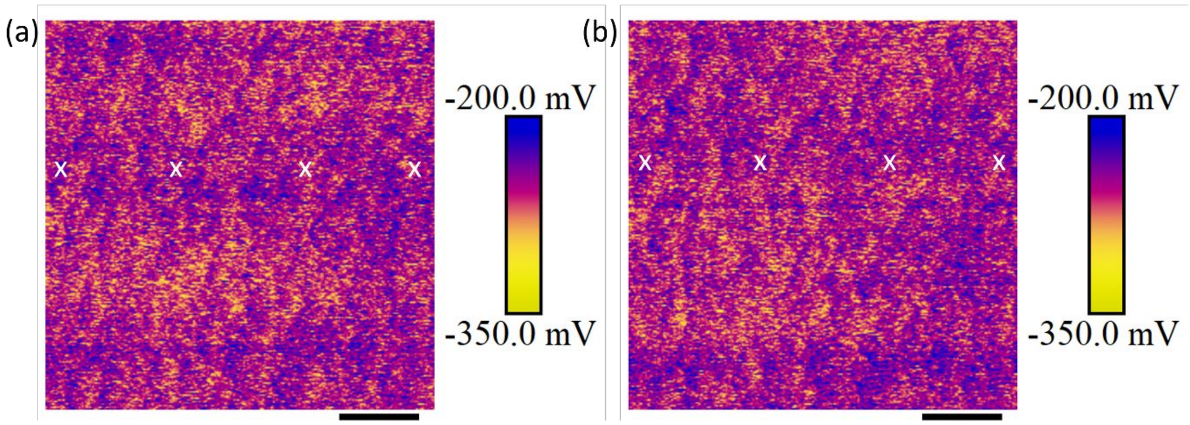

Figure S7. KPFM image (a) before and (b) after SS-PFM measurements of BFO thin film. SS-PFM measurements were performed at 4 different points, as displayed in the KPFM image. Here the PFM images are taken in 1 × 1 um^2 area.

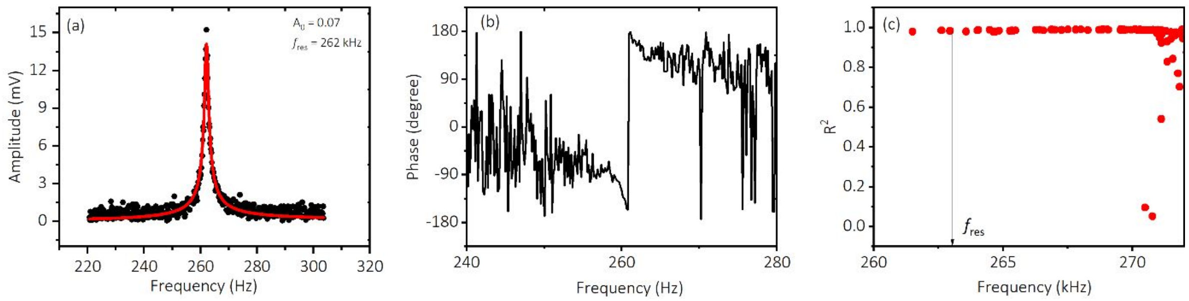

Figure S8. (a) SHO fitting of amplitude, (b) phase sweep with frequency, and (c) R^2 value with frequency for BTO.

Table S1: Summary of most influential factors which manipulate the true PFM results and their possible solutions.

**Factors contributing to the artefacts in true PFM response**	**A possible way to remove the artefacts**
A stiffer cantilever (high spring constant) could lead to misorientation of the phase loop.	A soft cantilever with 3-6 N/m spring constant is recommended.[1]
Low signal to noise ratio and asymmetric amplitude response at the contact resonance frequency.	Perform resonance enhanced amplitude sweeping and fit the amplitude using a SHO function.[1]
Parasitic phase offset arise from extrinsic factors.	Phase calibration is required to remove the phase offset before measurements. The detailed process is described in this work to identify and remove the parasitic phase offset (Figure 1).
Electrostatic interaction between the tip and sample. This arises from the inherent surface charge present on the ferroelectric sample.	(*i*) Phase and amplitude loop measurements should be performed in the bias off condition. (*ii*) Additional read voltage (equivalent to surface potential measured from KPFM) needs to be applied during the measurement to the tip to balance the surface charge on the sample. This is demonstrated in this work (Figures 2 and 3)

Crosstalk between the topography and PFM response in contact resonance mode	Adjustment in the set point (applied force on the sample) could help to minimize the crosstalk.[2]

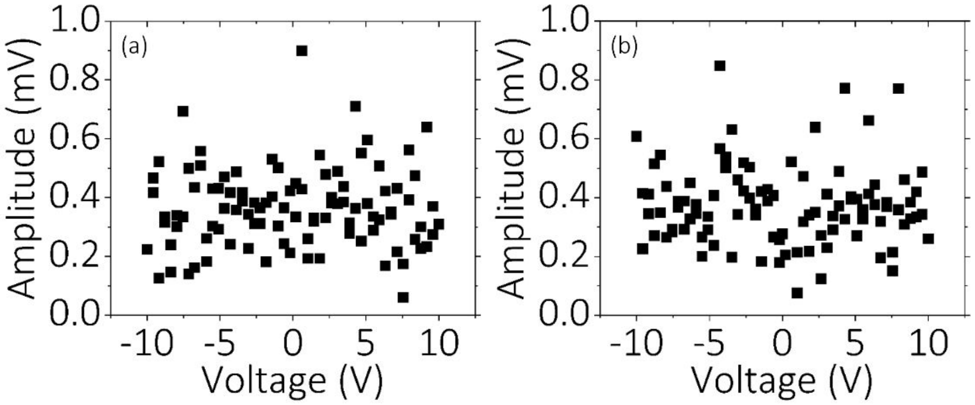

Figure S9. Amplitude versus voltage measured 50 nm away from the surface for (a) BFO and (b) BTO samples.

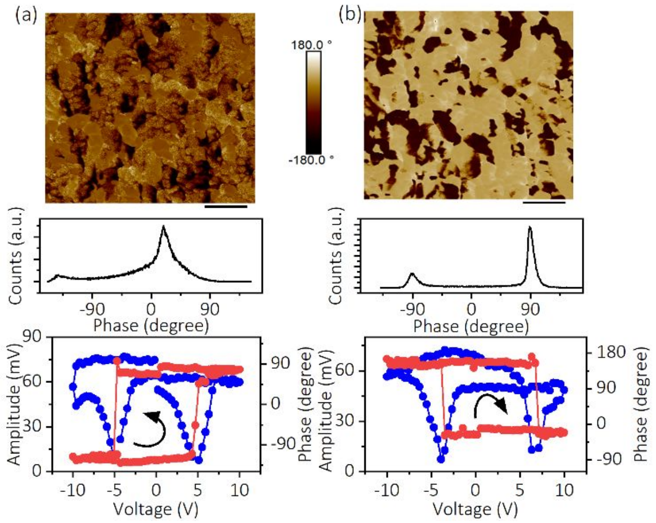

Figure S10. Phase image, domain distribution and phase-amplitude curve of BFO thin film represented in column (a) measured with uncalibrated and column (b) calibrated conditions. Here the PFM images are taken in 2 × 2 um^2 area.

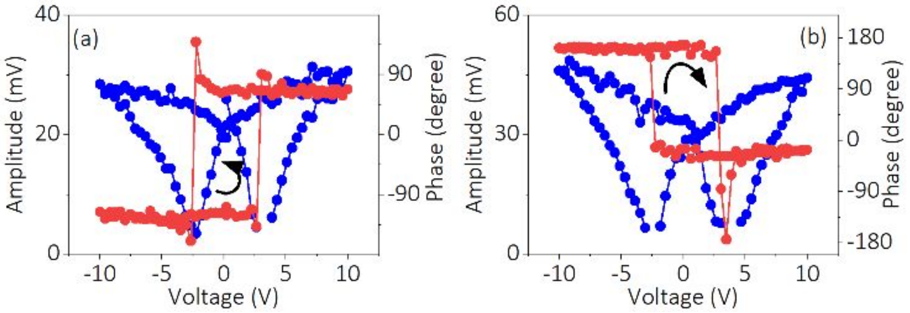

Figure 11. Phase-amplitude curve of BTO thin film measured with (a) uncalibrated and (b) calibrated conditions.

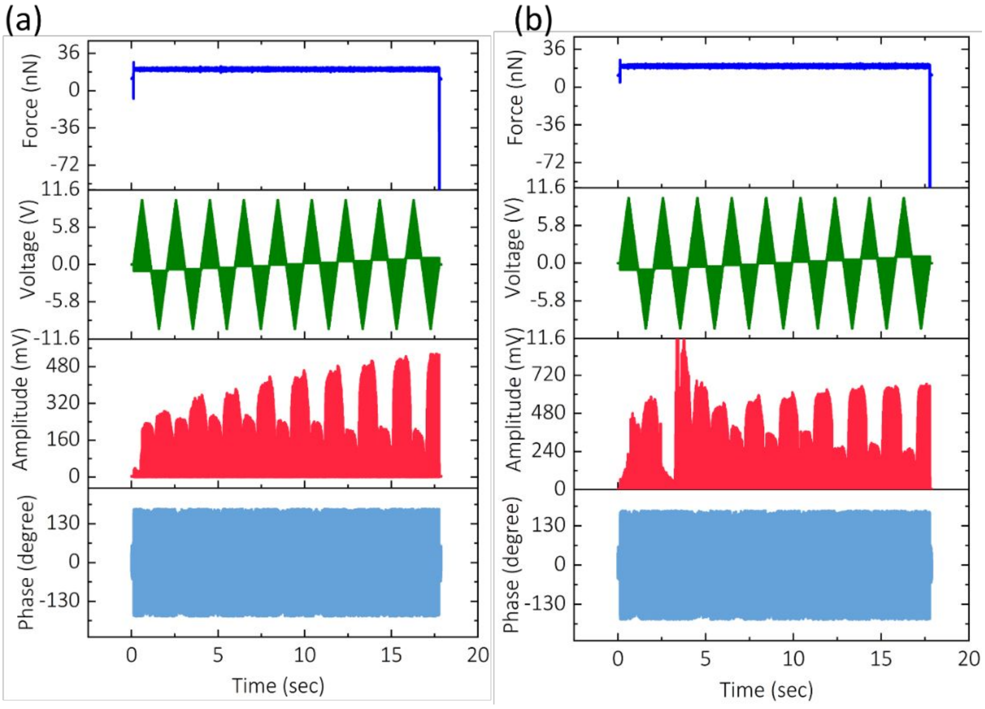

Figure S12. Force, voltage, amplitude and phase versus measurement time are plotted for BFO thin film at two different pixels. (a) pixel_1 and (b) pixel_2.

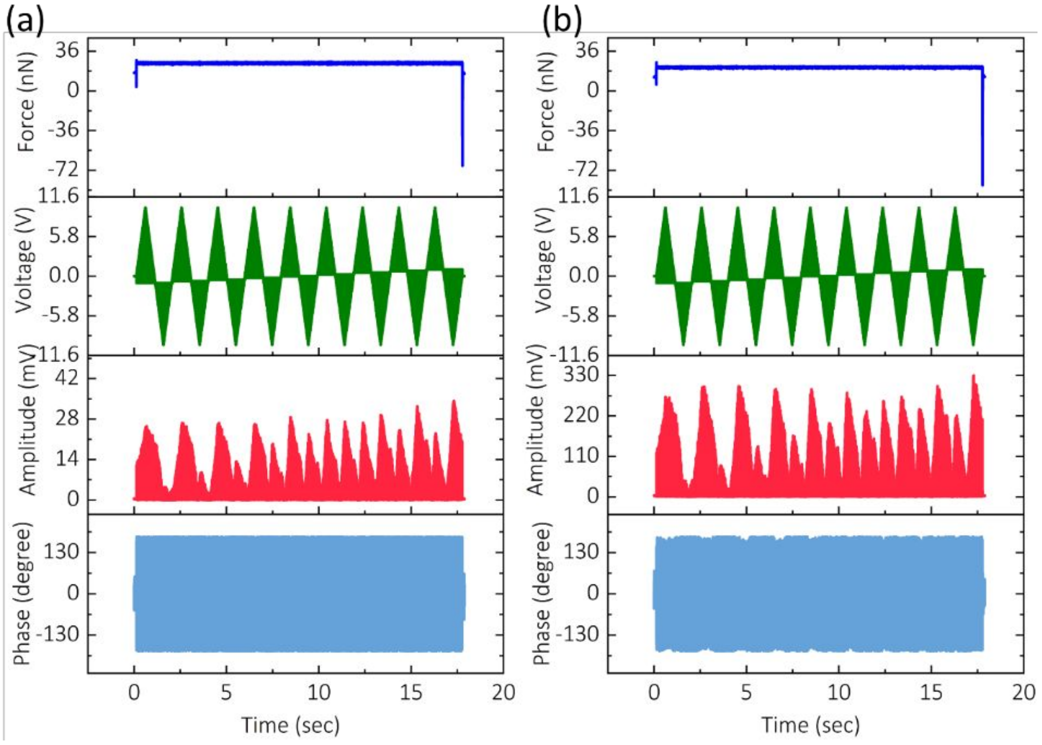

Figure S13. Force, voltage, amplitude and phase versus measurement time are plotted for BTO crystal at two different pixels. (a) pixel_1 and (b) pixel_2.

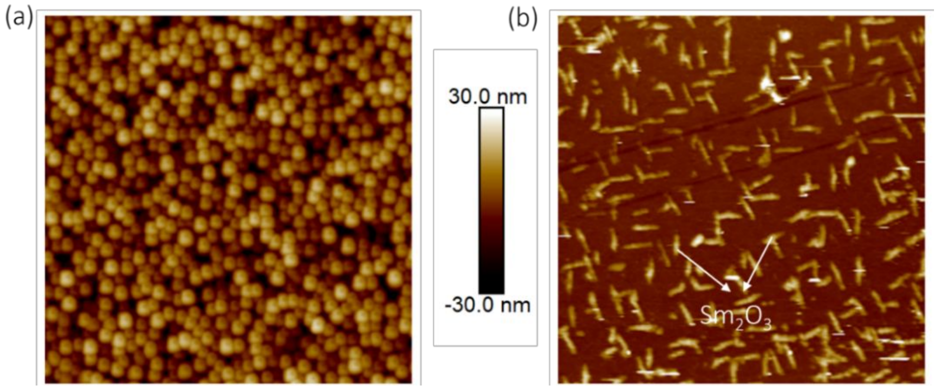

Figure S14: Topography image of 12 × 12 um^2 area of (a) BTO and (b) BTO:SmO thin films. In (b) the dark contrast area corresponds to the BTO matrix while the bright features consist of SmO. The features are ~900 nm long and ~100 nm wide, however the smaller SmO pillars (~20 nm) are also grown in the BTO matrix[3], but these are not visible at this scale.

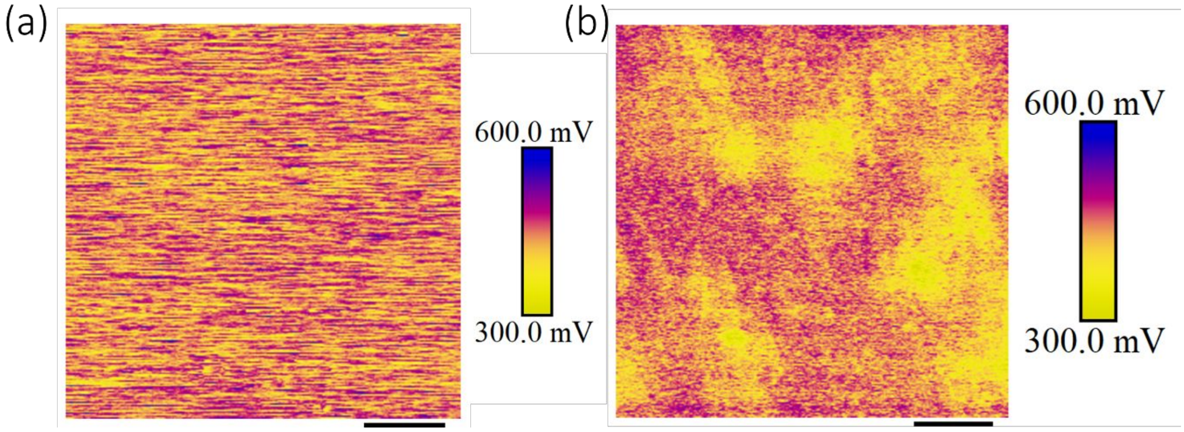

Figure S15: KPFM image of (a) BTO and (b)BTO:SmO nanocomposites. Here the KPFM images are taken in 2 × 2 um^2 area.

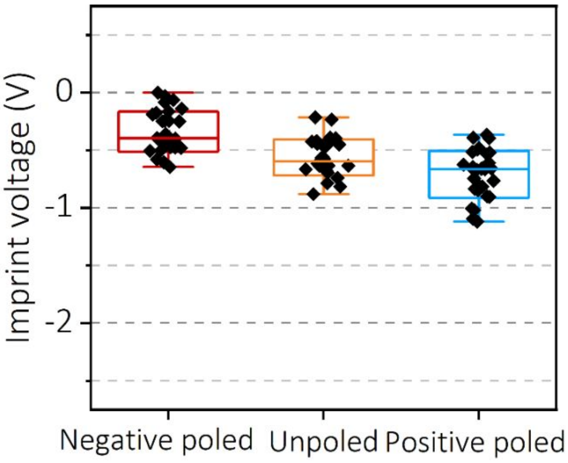

Figure S16: Imprint voltage extracted from BTO:MgO sample in the unpoled, positive, and negative poled conditions at $V_r = 0$ V.

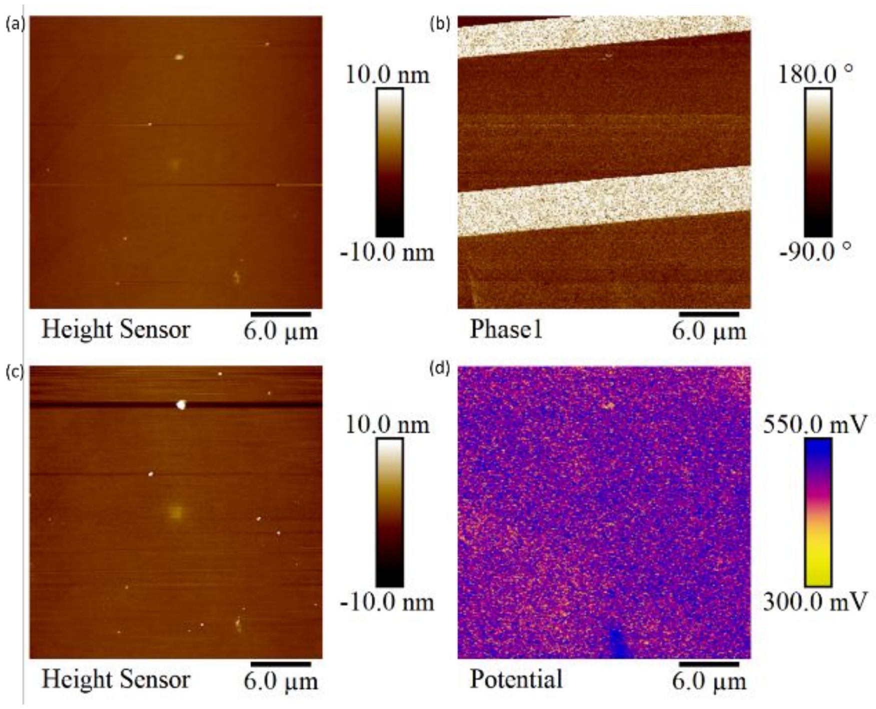

Figure S17. (a) topography, (b) PFM phase of BTO crystal measured by PFM technique. (c) topography and (d) surface potential measured using the KPFM technique.

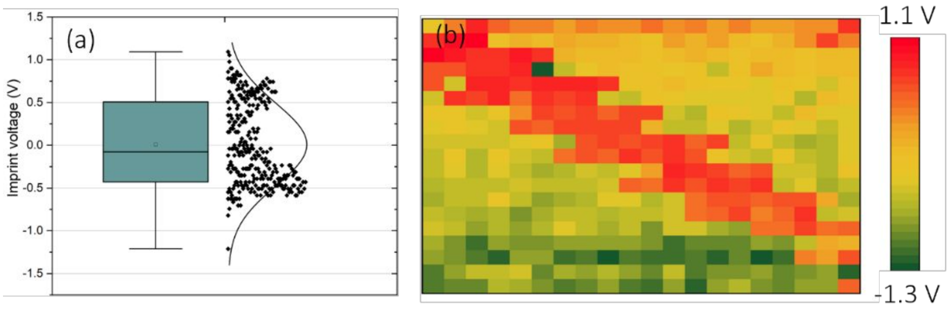

Figure S18. (a) Imprint voltage distribution of the BTO crystal. (b) The imprint voltage map in the same area.

In BTO crystal, the up and down domain shows positive and negative imprint voltage (Fig 7). However, the average response shows zero imprint voltage, which is normally observed when we perform macroscopic measurements. The variation in the imprint value for the up and down-oriented domain is only noticed when the calibrated phase amplitude response is obtained from pixels-by-pixel measurements.

REFERENCES

1. Neumayer, S. M.; Saremi, S.; Martin, L. W.; Collins, L.; Tselev, A.; Jesse, S.; Kalinin, S. V.; Balke, N. Piezoresponse Amplitude and Phase Quantified for Electromechanical Characterization J. Appl. Phys. 2020, 128, 171105.
2. Rodriguez, B. J.; Callahan, C.; Kalinin, S. V.; Proksch, R. Dual-frequency resonance-tracking atomic force microscopy Nanotechnology 2007, 18, 475504.
3. Harrington, S. A.; Zhai, J.; Denev, S.; Gopalan, V.; Wang, H.; Bi, Z.; Redfern, S. A. T.; Baek, S.-Hyub; Bark, C. W.; Eom, C.-Beom; Jia, Q.; Vickers, M. E.; MacManus-Driscoll, J. L. Thick Lead-Free Ferroelectric Films With High Curie Temperatures Through Nanocomposite-Induced Strain Nat. Nanotechnol. 2011, 6, 491.
